# Supplementary material for: Comprehensive Genomic Characterization of Fifteen Early-Onset Lynch-Like Syndrome Colorectal Cancers
Source: Cancers (Basel). 2021 Mar 12;13(6):1259. doi: 10.3390/cancers13061259 (PMC7999079; doi:10.3390/cancers13061259)
Supplement: Supplementary file 1 [file cancers-13-01259-s001.pdf]

cancers

MDPI

Supplementary Materials: Comprehensive Genomic Characterization of Fifteen Early-Onset Lynch-Like Syndrome Colorectal Cancers

Mariano Golubicki, Marcos Díaz-Gay, Laia Bonjoch, Sebastià Franch-Expósito, Jenifer Muñoz, Miriam Cuatrecasas, Teresa Ocaña, Soledad Iseas, Guillermo Mendez, Marcela Carballido, Juan Robbio, Daniel Cisterna, Enrique Roca, Antoni Castells, Francesc Balaguer, Sergi Castellví-Bel and Marina Antelo

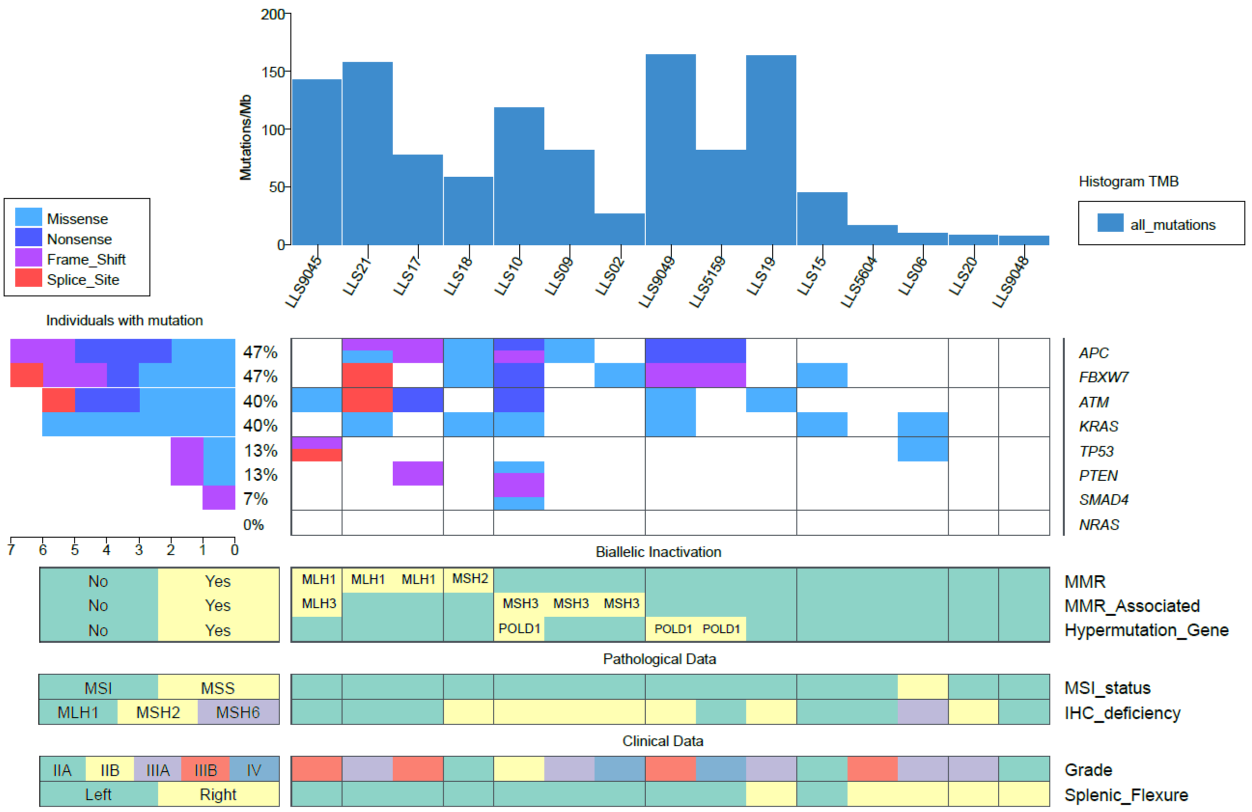

Figure S1. Additional most common somatic mutated genes in colorectal cancer.

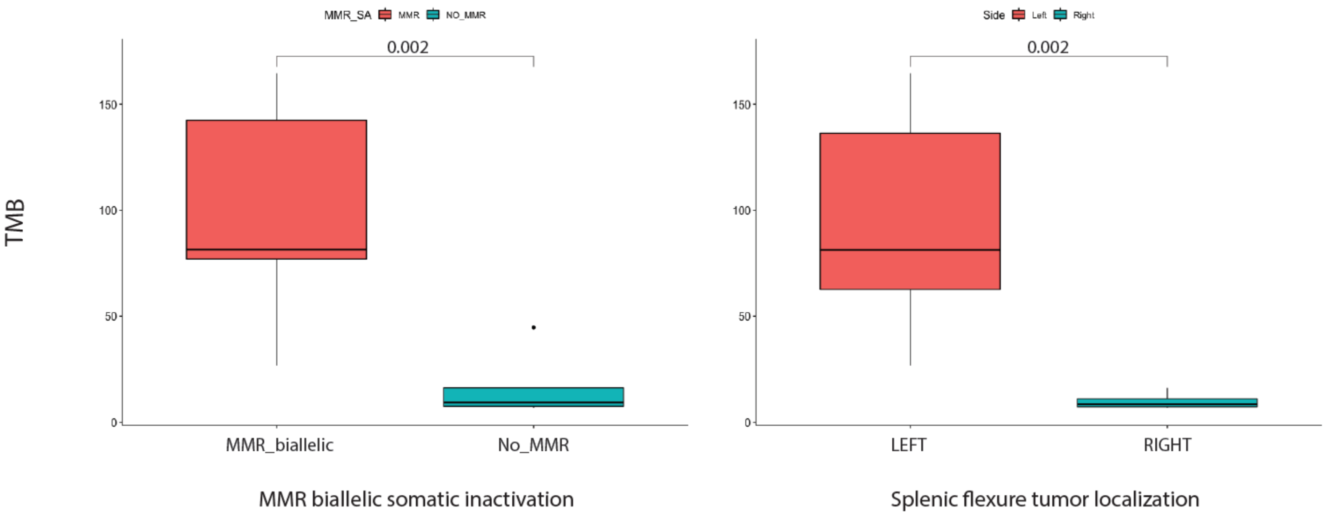

Figure S2. TMB comparison between biallelic somatic inactivations patients and with those who are not biallelic inactivated.

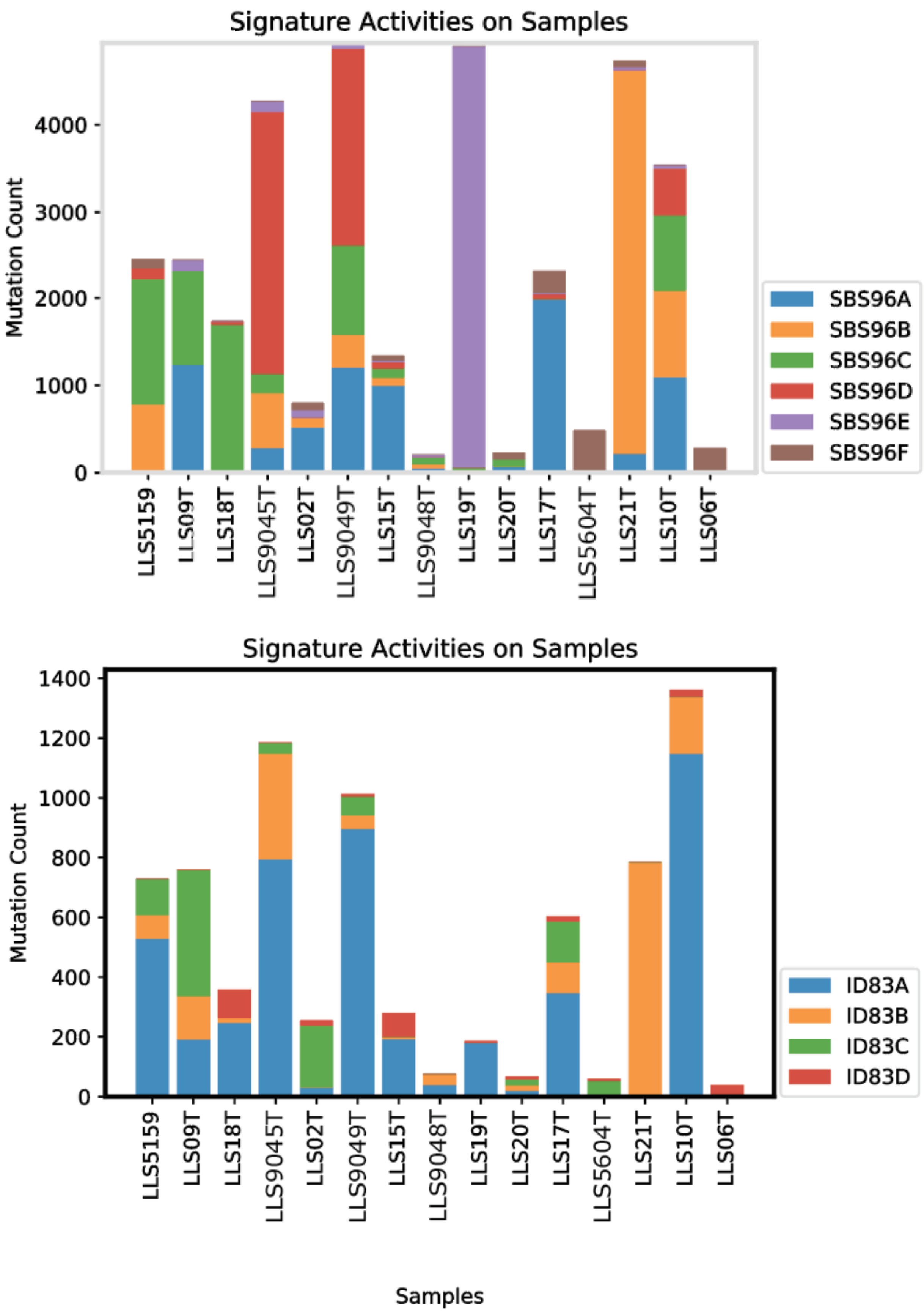

Figure S3. Single Base Substitution and InDels De novo mutational signatures.

Table S1. CRC Patients from CBioPortal with MSH3 truncating variants and no other MMR alterations showing microsatellite instability.

SAMPLE ID	STUDY ORIGIN IN CBIOPORTAL	MSH3 VARIANTS	ANNOTATION	MSI STATUS	MUTATION COUNT
11CO033	Colon Cancer (CPTAC-2 Prospective, Cell 2019)	K383Rfs*32	OncoKB: Likely Oncogenic, level NA;CIViC: NA;MyCancerGenome: not present;CancerHotspot: no;3DHotspot: no	MSI	1810
11CO036	Colon Cancer (CPTAC-2 Prospective, Cell 2019)	S1022*	OncoKB: Likely Oncogenic, level NA;CIViC: NA;MyCancerGenome: not present;CancerHotspot: no;3DHotspot: no	MSI	1805
11CO061	Colon Cancer (CPTAC-2 Prospective, Cell 2019)	K383Rfs*32	OncoKB: Likely Oncogenic, level NA;CIViC: NA;MyCancerGenome: not present;CancerHotspot: no;3DHotspot: no	MSI	736
TCGA-AA-A00A	Colorectal Adenocarcinoma (TCGA, Nature 2012)	K381Gfs*20	OncoKB: Likely Oncogenic, level NA;CIViC: NA;MyCancerGenome: not present;CancerHotspot: no;3DHotspot: no	MSI	324
TCGA-AA-A01Q	Colorectal Adenocarcinoma (TCGA, Nature 2012)	K381Gfs*20	OncoKB: Likely Oncogenic, level NA;CIViC: NA;MyCancerGenome: not present;CancerHotspot: no;3DHotspot: no	MSI	595
TCGA-AA-3492	Colorectal Adenocarcinoma (TCGA, PanCancer Atlas)	N861Mfs*6	OncoKB: Likely Oncogenic, level NA;CIViC: NA;MyCancerGenome: not present;CancerHotspot: no;3DHotspot: no	MSI	no data
TCGA-AA-3663	Colorectal Adenocarcinoma (TCGA, PanCancer Atlas)	N1020Ifs*40	OncoKB: Likely Oncogenic, level NA;CIViC: NA;MyCancerGenome: not present;CancerHotspot: no;3DHotspot: no	no data	1937
coadread_dfci_2016_60	Colorectal Adenocarcinoma (DFCI, Cell Reports 2016)	K383Rfs*32	OncoKB: Likely Oncogenic, level NA;CIViC: NA;MyCancerGenome: not present;CancerHotspot: no;3DHotspot: no	MSI	1257
coadread_dfci_2016_116	Colorectal Adenocarcinoma (DFCI, Cell Reports 2016)	Q1058*	OncoKB: Likely Oncogenic, level NA;CIViC: NA;MyCancerGenome: not present;CancerHotspot: no;3DHotspot: no	MSS	4474
coadread_dfci_2016_261	Colorectal Adenocarcinoma (DFCI, Cell Reports 2016)	K383Rfs*32	OncoKB: Likely Oncogenic, level NA;CIViC: NA;MyCancerGenome: not present;CancerHotspot: no;3DHotspot: no	MSI	1062
coadread_dfci_2016_407	Colorectal Adenocarcinoma (DFCI, Cell Reports 2016)	K383Rfs*32	OncoKB: Likely Oncogenic, level NA;CIViC: NA;MyCancerGenome: not present;CancerHotspot: no;3DHotspot: no	MSI	535
coadread_dfci_2016_1212	Colorectal Adenocarcinoma (DFCI, Cell Reports 2016)	K383Rfs*32	OncoKB: Likely Oncogenic, level NA;CIViC: NA;MyCancerGenome: not present;CancerHotspot: no;3DHotspot: no	MSI	1739
coadread_dfci_2016_2227	Colorectal Adenocarcinoma (DFCI, Cell Reports 2016)	X303_splice	OncoKB: Likely Oncogenic, level NA;CIViC: NA;MyCancerGenome: not present;CancerHotspot: no;3DHotspot: no	MSI	588

Table S2. Comparative studies showing clinical and molecular differences in published cohorts.

Author	year	Number of CRC LLS patients	Mean age at CRC diagnosis (years old)	Patients with double somatic alterations in MMR genes (%) of total cohort	Number of patients with CRC≤40	Mean age at CRC diagnosis (years old)	Patients with Double somatic alterations in MMR genes (%) of patients with CRC≤40
Sourouille	2013	17	54	24%	3	28	0%
Haraldsottir	2014	15	54	93%	4	34	100%
Mensenkamp	2014	23	47	48%	5	33	60%
Geurts-Giele W	2014	33	55	51%	5	35	0%
Porkka	2019	14	65	79%	1	38	100%
Xicola	2019	11	63	54%	0	-	-
Golubicki	2020	15	30	33%	15	30	33%

Table S3. Additional quality sequencing data from Table 2 variants.

Sample ID	Gene name	HGVS.c	HGVS.p	Variant impact	TumorAD	TumorDP	Tumor AF
**Biallelic somatic variants**							
**LLS02**	***MSH3***	**c.423C>A**	**p.Cys141***	**stop_gained**	**4,6**	**10**	**0,6**
**LLS09**	***MSH3***	**c.1148delA**	**p.Lys383fs**	**frameshift_variant**	**7,6**	**13**	**0,4615**
**LLS10**	***MSH3***	**c.1148delA**	**p.Lys383fs**	**frameshift_variant**	**28,43**	**71**	**0,6056**
LLS10	*POLD1*	c.583C>T	p.Arg195*	stop_gained	119,68	187	0,3636
LLS10	*POLD1*	c.2959delG	p.Asp987fs	frameshift_variant	85,56	141	0,3972
LLS17	*MLH1*	c.129dupA	p.Ser44fs	frameshift_variant	25,7	32	0,2188
LLS17	*MLH1*	c.1831delA	p.Ile611fs	frameshift_variant	52,26	78	0,3333
LLS18	*MSH2*	c.2251G>T	p.Gly751*	stop_gained	87,15	102	0,1471
LLS18	*MSH2*	c.2634+1G>A	-	splice_variant	73,17	90	0,1889
LLS21	*MLH1*	c.199G>A	p.Gly67Arg	missense_variant	129,2	149	0,1342
LLS21	*MLH1*	c.602delT	p.Val201fs	frameshift_variant	134,43	177	0,2429
LLS5159	*POLD1*	c.1562G>A	p.Arg521Gln	missense_variant	80,23	103	0,2233
LLS5159	*POLD1*	c.3047G>A	p.Arg1016His	missense_variant	57,21	78	0,2692
LLS9045	*MLH3*	c.3694C>T	p.Arg1232Cys	missense_variant	55,22	77	0,2857
**LLS9045**	***MLH3***	**c.1924T>C**	**p.Phe642Leu**	**missense_variant**	**37,32**	**69**	**0,4638**
LLS9045	*MLH1*	c.588delA	p.Lys196fs	frameshift_variant	25,7	32	0,2188
LLS9045	*MLH1*	c.1489delC	p.Arg497fs	frameshift_variant	119,32	151	0,2119
**LLS9049**	***POLD1***	**c.2959delG**	**p.Asp987fs**	**frameshift_variant**	**50,22**	**72**	**0,3056**
**Sample ID**	**Gene name**	**HGVS.c**	**HGVS.p**	**Variant impact**	**TumorAD**	**TumorDP**	**Tumor AF**
**Monoallelic somatic variants**							
LLS09	*MSH6*	c.3552G>A	p.Met1184Ile	missense_variant	19,8	27	0,2963
LLS10	*MSH6*	c.2875C>T	p.Arg959Cys	missense_variant	76,4	116	0,3448
LLS10	*POLE*	c.2091delC	p.Leu698fs	frameshift_variant	88,33	121	0,2727
LLS18	*MSH6*	c.3261dupC	p.Phe1088fs	frameshift_variant	324,47	371	0,1267
LLS19	*MSH2*	c.1225C>T	p.Gln409*	stop_gained	63,12	75	0,16
LLS19	*MSH6*	c.1993G>A	p.Glu665Lys	missense_variant	108,15	123	0,122
LLS21	*MSH3*	c.3356T>C	p.Leu1119Pro	missense_variant	30,5	35	0,1429
LLS21	*PMS2*	c.1239delA	p.Asp414fs	frameshift_variant	64,16	80	0,2
LLS21	*POLE*	c.1060A>G	p.Thr354Ala	missense_variant	49,12	61	0,1967
LLS9045	*MUTYH*	c.724C>T	p.Arg242Cys	missense_variant	58,33	91	0,3626
LLS9045	*POLD1*	c.735G>T	p.Glu245Asp	missense_variant	73,27	100	0,27
LLS9049	*MUTYH*	c.544C>T	p.Arg182Trp	missense_variant	99,15	114	0,1316
LLS9049	*MSH3*	c.433G>T	p.Ala145Ser	missense_variant	51,9	60	0,15
LLS9049	*POLE*	c.3176G>A	p.Arg1059His	missense_variant	97,24	121	0,1983

Table S4. Insight database classification from somatic MMR variants in Table 2.

InSight Classification for MMR variants				
**Biallelic Somatic Variants**				
**Patient ID**	**Gene Name**	**HGVS.c**	**HGVS.p**	**InSight Classification**
LLS17	*MLH1*	c.129dupA	p.Ser44fs	not present
**LLS17**	***MLH1***	**c.1831delA**	**p.Ile611fs**	**Class 5: Pathogenic**
LLS18	*MSH2*	c.2251G>T	p.Gly751*	not present
**LLS18**	***MSH2***	**c.2634+1G>T**	**-**	**Class 4: Likely Pathogenic**
**LLS21**	***MLH1***	**c.199G>A**	**p.Gly67Arg**	**Class 5: Pathogenic**
LLS21	*MLH1*	c.602delT	p.Val201fs	not present
**LLS9045**	***MLH1***	**c.588delA**	**p.Lys196fs**	**Class 5: Pathogenic**
LLS9045	*MLH1*	1849delC	p.Arg497fs	not present
**Monoallelic Somatic Variants**				
**Patient ID**	**Gene Name**	**HGVS.c**	**HGVS.p**	**InSight Classification**
LLS09	*MSH6*	c.3552G>A	p.Met1184Ile	not present
LLS10	*MSH6*	c.2875C>T	p.Arg959Cys	not present
**LLS18**	***MSH6***	**c.3261dupC**	**p.Phe1088fs**	**Class 5: Pathogenic**
LLS19	*MSH2*	c.1225C>T	p.Gln409*	not present
LLS19	*MSH6*	c.1993G>A	p.Glu665Lys	not present
LLS21	*PMS2*	c.1239delA	p.Asp414fs	not present

Table S5. ACMG classification criteria from Table 2 somatic variants.

Biallelic somatic variants

Sample ID	Gene name	HGVS.c	HGVS.p	Variant impact	gnomAD	ClinVar	COSMIC	ACMG classification criteria
**LLS02**	***MSH3***	**c.423C>A**	**p.Cys141***	**stop_gained**	**not present**	**not present**	**not present**	**Pathogenic (PVS1; PMS2; PP3)**
**LLS09**	***MSH3***	**c.1148delA**	**p.Lys383fs**	**frameshift_variant**	**1,64E-05**	**Pathogenic**	**COSM1438888**	**Pathogenic (PVS1; PS3; PP3; PP5)**
**LLS10**	***MSH3***	**c.1148delA**	**p.Lys383fs**	**frameshift_variant**	**1,64E-05**	**Pathogenic**	**COSM1438888**	**Pathogenic (PVS1; PS3; PP3; PP5)**
LLS10	*POLD1*	c.583C>T	p.Arg195*	stop_gained	0,0000647	Conflicting	not present	**Benign (PVS1; PP3; BS1, BS2)**
LLS10	*POLD1*	c.2959delG	p.Asp987fs	frameshift_variant	0,000057209	not present	COSM3686158	**Likely Pathogenic (PVS1; PM2)**
LLS17	*MLH1*	c.129dupA	p.Ser44fs	frameshift_variant	not present	Pathogenic	not present	**Pathogenic (PVS1; PM2; PP3; PP5)**
LLS17	*MLH1*	c.1831delA	p.Ile611fs	frameshift_variant	not present	not present	not present	**Pathogenic (PVS1; PM2; PP3)**
LLS18	*MSH2*	c.2251G>T	p.Gly751*	stop_gained	not present	not present	not present	**Pathogenic (PVS1; PM2; PP3)**
LLS18	*MSH2*	c.2634+1G>A	-	splice_variant	not present	Likely_pathogenic	not present	**Pathogenic (PVS1; PM2; PP3; PP5)**
LLS21	*MLH1*	c.199G>A	p.Gly67Arg	missense_variant	not present	Pathogenic	COSM1422567	**Pathogenic (PS1; PM1; PM2; PM5; PP2; PP3; PP5)**
LLS21	*MLH1*	c.602delT	p.Val201fs	frameshift_variant	not present	not present	not present	**Likely Pathogenic (PVS1; PM2)**
LLS5159	*POLD1*	c.1562G>A	p.Arg521Gln	missense_variant	0,000126296	VUS	not present	**Benign (PP3; BS1; BS2; BP1)**
LLS5159	*POLD1*	c.3047G>A	p.Arg1016His	missense_variant	not present	VUS	COSM7587416	**VUS (PM2; PP3; BP1)**
LLS9045	*MLH3*	c.3694C>T	p.Arg1232Cys	missense_variant	3,25E-05	VUS	not present	**VUS (PP3; BP1)**
**LLS9045**	***MLH3***	**c.1924T>C**	**p.Phe642Leu**	**missense_variant**	**not present**	**not present**	**not present**	**Likely Benign (PM2; BP1; BP4)**
LLS9045	*MLH1*	c.588delA	p.Lys196fs	frameshift_variant	4,06E-06	Pathogenic	not present	**Pathogenic (PVS1; PM2; PP3; PP5)**
LLS9045	*MLH1*	c.1489delC	p.Arg497fs	frameshift_variant	4,06E-06	not present	COSM1422596	**Pathogenic (PVS1; PM2; PP3; PP5)**
**LLS9049**	***POLD1***	**c.2959delG**	**p.Asp987fs**	**frameshift_variant**	**0,000057209**	**not present**	**COSM3686158**	**Likely Pathogenic (PVS1; PM2)**

Monoallelic somatic variants

Sample ID	Gene name	HGVS.c	HGVS.p	Variant impact	gnomAD	ClinVar	COSMIC	ACMG classification criteria
LLS09	*MSH6*	c.3552G>A	p.Met1184Ile	missense_variant	4,07E-06	not present	not present	**VUS (PM2; PP3)**
LLS10	*MSH6*	c.2875C>T	p.Arg959Cys	missense_variant	1,22E-05	VUS	not present	**VUS (PM2; PP3)**
LLS10	*POLE*	c.2091delC	p.Leu698fs	frameshift_variant	8,21E-06	VUS	COSM4612998	**Pathogenic (PVS1; PM2; PP3)**
LLS18	*MSH6*	c.3261dupC	p.Phe1088fs	frameshift_variant	0,00003449	Pathogenic	COSM13394	**Pathogenic (PVS1; PM2; PP3; PP5)**
LLS19	*MSH2*	c.1225C>T	p.Gln409*	stop_gained	not present	Pathogenic	COSM7508782	**Pathogenic (PVS1; PM2; PP3; PP5)**
LLS19	*MSH6*	c.1993G>A	p.Glu665Lys	missense_variant	4,07E-06	not present	not present	**VUS (PM2; PP3)**
LLS21	*MSH3*	c.3356T>C	p.Leu1119Pro	missense_variant	not present	not present	not prensent	**VUS (PM2; PP3)**
LLS21	*PMS2*	c.1239delA	p.Asp414fs	frameshift_variant	not present	not present	COSM150905	**Pathogenic (PVS1; PM2; PP3)**
LLS21	*POLE*	c.1060A>G	p.Thr354Ala	missense_variant	not present	not present	not present	**Likely Benign (PM2; BP1; BP4)**
LLS9045	*MUTYH*	c.724C>T	p.Arg242Cys	missense_variant	5,30E-05	Pathogenic	COSM6954579	**Pathogenic (PM1; PM2; PM5; PP2; PP3; PP5)**
LLS9045	*POLD1*	c.735G>T	p.Glu245Asp	missense_variant	not present	not present	not present	**Likely Benign (PM2; BP1; BP4)**
LLS9049	*MUTYH*	c.544C>T	p.Arg182Trp	missense_variant	1,22E-05	not present	COSM6922477	**Pathogenic (PM1; PM2; PM5; PP2; PP3; PP5)**
LLS9049	*MSH3*	c.433G>T	p.Ala145Ser	missense_variant	not present	VUS	not present	**VUS (PM2; BP4)**
LLS9049	*POLE*	c.3176G>A	p.Arg1059His	missense_variant	1,22E-05	not present	COSM6965827	**VUS (PM2; PP3; BP1)**
